# Particle Agglomeration of Acid-Modified Tapioca Starches: Characterization and Use as Direct Compression Fillers in Tablets

**DOI:** 10.3390/pharmaceutics14061245

**Published:** 2022-06-12

**Authors:** Chaipat Siriwachirachai, Thaned Pongjanyakul

**Affiliations:** Division of Pharmaceutical Technology, Faculty of Pharmaceutical Sciences, Khon Kaen University, Khon Kaen 40002, Thailand; chaipatsiriwa@gmail.com

**Keywords:** tapioca starch, agglomeration, acid modification, direct compression filler, particle flowability, tablets

## Abstract

Acid-modified tapioca starches (AMTSs) possessed good compressibility but showed poor particle flowability for preparing tablets by the direct compression method. The aims of this work were to prepare and characterize AMTS agglomerates using polyvinylpyrrolidone (PVP) as an agglomerating agent. The dilution potential and stability studies of the AMTS agglomerates were investigated. The results showed that particle enlargement of TS and AMTS could be achieved via agglomeration using PVP. The thermal behavior and molecular interaction of the agglomerates were revealed using DSC and FTIR spectroscopy, respectively. An increase in PVP concentrations resulted in greater particle strength of the TS agglomerates and a higher acid concentration for modification enhanced the strength of the AMTS agglomerates. All agglomerates presented good particle flowability. Moreover, the AMTS agglomerates provided higher compressibility hardness than the TS agglomerates. The addition of PVP could extend the disintegration time and slow drug dissolution from the agglomerate tablets. The humidity of the storage conditions influenced the thickness and hardness of the AMTS agglomerate tablets, and good physical and chemical stability of the tablets was obtained under ambient conditions and in the refrigerator. Furthermore, the AMTS agglomerates displayed good carrying capacity and possessed desirable characteristics for use in direct compression tablets.

## 1. Introduction

Pharmaceutical products are an indispensable requisite for the world’s population for the diagnosis and treatment of diseases and the promotion of well-being. Among those formulations, solid dosage formulations, particularly tablets, have become high in demand given their ease of delivery, economy, convenient administration, and prolonged drug stability [1]. Tablets generally have compressed drug powder and other inactive excipients and diluents confined within a limited volume, leading to the formation of a compacted solid. Direct compression is an ideal process for the formulation of tablets with uncomplicated steps, lower costs, and shorter time consumption [2]. Diluents or fillers used are important components to achieve good tablet performance, which is dependent on the characteristics of the materials, such as particle size uniformity, particle deformation, and stability [3]. Moreover, they are necessary to offer good flowability and compressibility [4,5]. Thus, it is interesting to seek novel materials or to modify a traditional material for use as a direct compression filler.

Starch, a biopolysaccharide, has been widely employed in pharmaceutical products because of its biodegradable and biocompatible properties [6,7]. The starch components are amylose and amylopectin. Amylose is a linear glucose chain linked by α-1-4 glycosidic bonding, whereas amylopectin partly provides α-1-6 glycosidic linkages to form branch chains [8]. Tapioca starch (TS) was obtained from the root of cassava (*Manihot esculenta* (L.) Crantz) in the family Euphoriaceae [9]. TS is one of the choices for pharmaceuticals that provides a clear paste, bland flavor, and low percentage of lipids and protein [10]. In addition, it can be applied as a diluent, binder, and disintegrant in tablet formulations. However, native TS could not offer sufficient flowability and compressibility for use as a direct compression filler [11], thus requiring starch modification to be performed. Acid treatment is one of the popular approaches for TS modification because it is performed using uncomplicated steps with simple instruments [12]. After acid hydrolysis, an alkali solution was added to the treated starch for the neutralization reaction [13,14]. Salt formation during neutralization is essential to complete removal because it influences the swelling of starches [15,16]. Spray drying at high temperature was employed for the drying process, resulting in an improvement in flowability [17] and compressibility [14,17]. Unfortunately, the drying process at high temperature caused a partial gelatinization of TS, leading to a change in the starch granule morphology and a decrease in the crystallinity [18].

Recently, the intrinsic physicochemical properties of acid-modified tapioca starches (AMTSs) prepared using water washing to eliminate acid residues and using tray drying at temperatures lower than the gelatinization temperature were reported [19]. The AMTSs treated with hydrochloric acid solution had higher temperature and enthalpy of gelatinization and greater percent relative crystallinity than the native TS. Furthermore, better compressibility of the AMTS was obtained when compared with the native TS. However, poor flowability of the AMTS was also found. Therefore, it is of interest to enhance the flowability of AMTSs by a size enlargement approach [20]. Particle agglomeration by adding an agglomerating agent (binder) was suitable for increasing the particle size of the AMTS because this method was not complicated, and the equipment used was similar to those for preparing tablets by wet granulation. Agglomeration of powders involves many types of bonding, such as liquid bridges, solid bridges, and interlocking bonds [21], to achieve particle size enlargement. This approach was successful in preparing particle agglomerates of composite materials using polyvinylpyrrolidone (PVP) as an agglomerating agent for direct compression tablets [5].

Therefore, the objectives of this work were to prepare and characterize AMTS agglomerates using PVP as an agglomerating agent and investigate the effect of PVP content on the characteristics of TS agglomerates as a control in this study. The thermal behavior, molecular interaction of the components, particle morphology, flowability, and compressibility of the TS and AMTS agglomerates were examined. Moreover, tablet characteristics, dilution potential, and stability in an accelerated condition of the agglomerates were also evaluated, in which the AMTS agglomerates may be applied as a direct compression filler for tablets.

## 2. Materials and Methods

### 2.1. Materials

Tapioca starch (TS) was purchased from Kaensiri Starch Co., Ltd. (Khon Kaen, Thailand). Acetaminophen (ACT) (K Sciences Center and Medical Ltd., Part., Khon Kaen, Thailand), propranolol hydrochloride (PPN) (Changzhou Yabang Pharmaceutical Co., Ltd., Changzhou, Jiangsu, China), polyvinylpyrrolidone K30 (PVP) (Union Sciences Trading Co., Ltd., Khon Kaen, Thailand), Starch 1500^®^ (Colorcon Ltd., Dartford, UK), and magnesium stearate (Mallinckrodt Inc., St. Louis, MO, USA) were employed in this study. All other reagents were of analytical grade and used as received.

### 2.2. Preparation of TS and AMTS Agglomerates

Native starch with a solid content of 25% *w*/*v* was suspended in 0.0, 0.1, 0.5, or 1.0 N HCl with constant stirring using a magnetic stirrer at room temperature (26–28 °C) for 48 h. After that, the wet starch cake was collected using negative pressure filtration with filter paper (Whatman^®^ No. 4) and washed using distilled water until the filtrate pH was 6–7. Before incorporating PVP, the wet cake of the starches was accurately weighed and dried at 55 °C in a hot air oven to achieve a constant weight, and the percent dry starch content in the wet starch cake was then computed. PVP in the concentration range of 0–6% *w/w* of the dry starch content was added to induce an agglomeration of starches. The PVP was dispersed in distilled water and was blended homogeneously into the wet starch cake using a mortar and pestle. Then, the wet mass obtained was pressed through a 1.7-mm sieve and dried at 55 °C in a hot air oven. Next, the dry particles were passed through a 150-μm sieve before particle size selection. The particles in the size range of 75–150 µm were selected and collected using a sieve analyzer, and the percent yield of the agglomerated particles was also computed. The TS and AMTS agglomerates were stored in a silica gel bead desiccator at room temperature (26–28 °C) prior to investigation.

### 2.3. Characterization of TS and AMTS Agglomerates

#### 2.3.1. Particle Morphology Studies

The particle morphology of the TS and AMTS agglomerates was investigated by scanning electron microscopy (SEM). The agglomerates were mounted onto a dummy using adhesive carbon tape. The samples were then coated with gold in a vacuum system and observed under a scanning electron microscope (Hitachi S-3000N, Tokyo, Japan).

#### 2.3.2. Differential Scanning Calorimetry (DSC)

The thermal behavior of the starch agglomerates was examined using DSC. A weighed sample was placed in a 40-μL aluminum dish without sealing. DSC thermograms were recorded in the temperature range of 30–400 °C at a heating rate of 10 °C min^−1^ (DSC822e, Mettler Toledo, Greifensee, Switzerland).

#### 2.3.3. Fourier Transform Infrared (FTIR) Spectroscopy

The crucial functional groups of the starch agglomerates were investigated by FTIR spectroscopy using the KBr disk method. Samples were ground with KBr pellets before pressing as a disk using a hydraulic press at 10 tons for 10 min. The disk was then placed in a sample holder and scanned at a resolution of 4 cm^−^^1^ from 4000 to 400 cm^−^^1^ (Spectrum One, Perkin Elmer, Norwalk, CT, USA).

#### 2.3.4. Particle Flowability Measurement

The flowability of the agglomerates was determined in terms of the angle of repose and Carr’s index. The angle of repose was measured by continuously pouring the agglomerates through a funnel onto a smooth surface, forming a fixed-height conical pile. The height (h) and radius (r) of the pile were noted, and the angle of repose (α) was calculated from the following equation:

$$\tan\alpha =\frac{h}{r}$$

For Carr’s index, agglomerates (10 g) were weighed and gently poured into a 50-mL cylinder. The initial volume of the agglomerates was recorded and calculated as a bulk density (D_B_). Then, the sample-containing cylinder was tapped 1.5 inches in height until the volume was constant. The final volume was recorded and computed as a tapped density (D_T_). Carr’s index was calculated using the following equation:

$$\mathrm{Carr}’s\;\mathrm{index}\;\left(\%\right)=\frac{D_{T}-D_{B}}{D_{T}}\times 100$$

#### 2.3.5. Particle Strength Determination

The strength of the agglomerates was measured a previously reported method [22,23]. The greater particle size of the agglomerates was employed. The wet mass of the TS or AMTS blended with PVP was passed through a 1.7-mm sieve and dried at 55 °C in a hot air oven. A texture analyzer (TA. XT plus, Stable Micro Systems, Godalming, UK) equipped with a 50-kg load cell with a 6-mm diameter cylindrical probe was utilized. Initially, the agglomerate was placed on the platform at room temperature. The probe was positioned to touch the surface of the agglomerate and then moved downward at a constant rate of 1.0 mm s^−^^1^. Next, the probe was removed automatically when the agglomerates were pressed and reduced to 50% of the original height. The force and percent displacement were plotted, and the maximum force at 50% displacement, representing the strength of the agglomerates, was reported.

### 2.4. Tablet Preparations

A tablet without drug was prepared by using a direct compression method. The TS or AMTS agglomerates were mixed thoroughly with magnesium stearate as a lubricant at a content of 1% *w/w* for 2 min. The 252.5 mg mixture was weighed and placed into a 10 mm-diameter flat-faced die and punched. Compression in the range of 4.9 to 12.3 MPa was applied using a hydraulic press (Model 3126, Shimadzu, Kyoto, Japan) without holding time. The tablets thus obtained were kept in a silica gel bead desiccator until characterization.

For PPN tablets, each tablet containing 40 mg of PPN and 210 mg of TS or AMTS agglomerates was blended in a rotomixer for 3 min. Then, 1% *w*/*w* magnesium stearate was added and mixed continuously for 2 min. The mixture (252.5 mg) was filled into a 10 mm-diameter flat-faced die, punched, and compressed at 12.3 MPa using a hydraulic press without holding time. The PPN tablets obtained were stored in a desiccator prior to testing.

To test the feasibility of starch agglomerates as a direct compression filler in scaled-up production, a dilution potential study was performed, and ACT was used as a model drug. Each tablet (350 mg) was composed of ACT with different contents (0, 10, 20, 30, and 40% *w*/*w*) and starch agglomerates. Both substances were mixed in a rotomixer for 10 min. Then, magnesium stearate (1% *w*/*w*) was added to the mixture, which was mixed again for 5 min. The mixtures were compressed using a single punch-tableting machine (Yeo Heng Co., Ltd., Bangkok, Thailand) with a 10-mm flat-faced die and punches. The batch size was 500 tablets. The thickness and weight of tablets were controlled in this study. The physical properties and drug dissolution of the tablets were evaluated.

### 2.5. Stability Study of Tablets

The tablets containing 20% ACT from the dilution potential study were employed in this study. The tablets were filled in a tightly closed plastic container and stored under ambient conditions (26 ± 2 °C, 52 ± 3% relative humidity (RH)), a refrigerator (2–8 °C, 20 ± 2% RH) and accelerated conditions (45 ± 1 °C, 75% RH). For the accelerated condition, they were placed in a desiccator containing saturated sodium chloride solution (75% RH), and the desiccator was stored in a hot air oven at 45 ± 1 °C. After storage (3 months), the tablets were collected and evaluated.

### 2.6. Tablet Evaluations

The thickness and hardness of the tablets were evaluated by using a Vernier caliper and a tablet hardness tester (Model 40-2100 VK200 VanKel^®^, Cary, NC, USA), respectively. The tablet disintegration time was determined by a basket rack assembly disintegration test apparatus (Model ZT-324, Erweka America Inc., Edison, NJ, USA) with a cover sieve. The media were 0.1 N HCl and pH 6.8 phosphate buffer at 37.0 ± 0.5 °C. Each tablet was placed into the basket without a dish. The disintegration time was recorded when the tablet was completely liberated from the screen of the basket.

For the dilution potential study, the weight variation, percent friability, and ACT content were also determined. Twenty tablets were weighed using an analytical balance. The results were expressed in terms of average weight with standard deviation, and the relative standard deviation (RSD) was calculated and reported. To test percent friability, excess powders were removed from the surface of the tablets before testing. At least 6.5 g tablets were accurately weighed and placed in the drum of a tablet friability instrument (Model 45-2200 VanKel^®^, Cary, NC, USA). Then, the drum containing the tablets was rotated 100 revolutions within 4 min. After that, the tablets were taken out, and excess dust was removed. The remaining weight of the tablets was accurately weighed, and the percent friability (weight loss) was calculated. Moreover, the ACT content of the tablets was determined by soaking the tablets in 100 mL of 0.1 N HCl for 1 h. The sample was filtered through a 0.45-µm cellulose acetate membrane. The clear solution was analyzed for ACT concentration using a UV–visible spectrophotometer (Model UV1201 Shimadzu, Kyoto, Japan) at a wavelength of 265 nm.

The drug dissolution study of tablets was characterized using a USP dissolution apparatus I (basket method) (Hanson Research 72RL, Chatsworth, CA, USA). The medium (750 mL) was 0.1 N HCl or pH 6.8 phosphate buffer, and a controlled temperature system (37.0 ± 1.0 °C) was used in this study. The basket was rotated at a rate of 50 revolutions min^−^^1^. The samples were collected and replaced with an equal volume of fresh medium at each designated time point. The drug concentrations were analyzed by a UV–visible spectrophotometer (Shimadzu UV1201, Japan) at a wavelength of 265 for ACT and 289 for PPN. To compare drug dissolution of the tablets, T_50__%_, the time to achieve dissolution of 50% of drug content, was calculated. Additionally, the similarity factor (f_2_) was used to measure the closeness of the drug dissolution profiles between the reference (tablets at the initial stage) and test samples (tablets stored under various conditions for 3 months). The similarity factor was computed using the equation [24,25] as follows:

$$\mathrm{Similarity}\;\mathrm{factor}\;\left(f_{2}\right)=50\cdot \log\left\{{\left[1+\frac{1}{n}\overset{n}{\underset{t=1}{\sum }}{\left(R_{t}-S_{t}\right)}^{2}\right]}^{-0.5}\times 100\right\}$$

where *n* is the number of time points and R_t_ and S_t_ are the percent drug dissolved in the reference and test samples at time t, respectively. The similarity of the two drug dissolution profiles occurs when the calculated f_2_ value is in the range of 50 to 100.

## 3. Results and Discussion

### 3.1. Appearance, Morphology and Strength of the Agglomerates

TS and AMTS agglomerates in the particle size range of 75–150 µm were successfully prepared in this study. The percent yield of the TS agglomerates increased with increasing PVP contents in the range of 0–4% *w*/*w*, but the 4 and 6% PVP gave similar percent yields of the TS agglomerates (Table 1). It can be observed that the use of 6% PVP showed some stickiness of the agglomerates at room temperature. This was due to the hygroscopic property of PVP [26]. For this reason, 4% *w*/*w* PVP was selected and used as the agglomerating agent for AMTS. The AMTS agglomerates resulting from higher HCl concentrations being used for acid modification presented higher yields of this particle size range when using the same PVP content (Table 1). The moisture content of all agglomerates was in the range of 9–12%, which was within the acceptable value of TSs [27].

**Figure 1:**
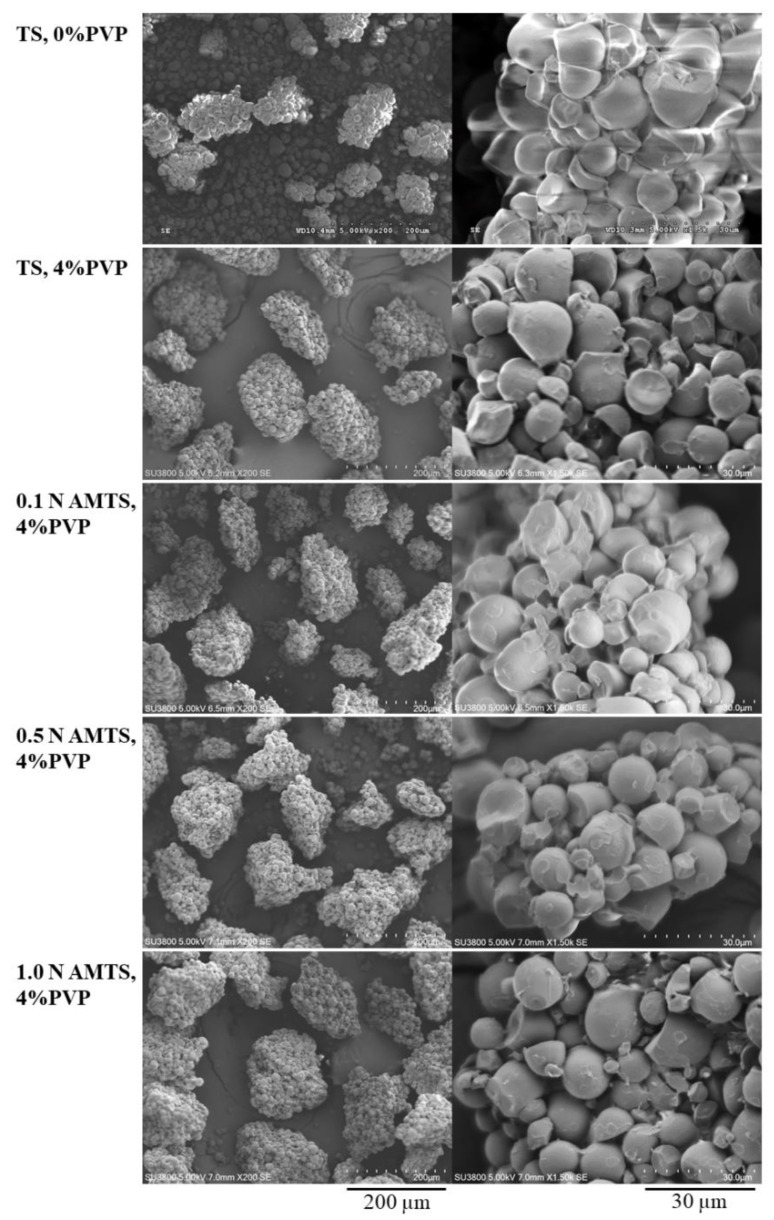
Particle morphology of TS and AMTS agglomerates.

Figure 1 illustrates the particle morphology of the agglomerates observed using SEM. The TS without PVP presented an aggregation of the starch granules. Incorporation of PVP resulted in a greater particle size of TS agglomerates. The AMTS agglomerates presented a particle morphology similar to that of the TS agglomerates with 4% PVP, although they were treated with different HCl concentrations. Additionally, the rough surface of the starch granules of the TS and AMTS agglomerates with 4% PVP was observed. This result occurred from the PVP thin films coated and attached to the surface of the starch granules during the agglomeration process.

**Figure 2:**
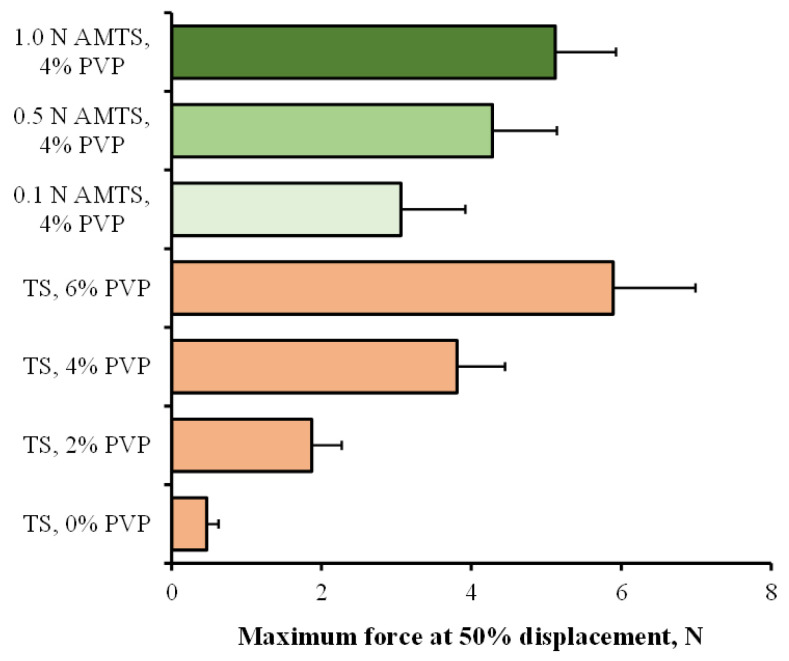
Maximum force at 50% displacement of TS and AMTS agglomerates. Each value is the mean ± S.D., *n* = 20.

The particle strength of the agglomerates was measured by using the maximum force at 50% displacement as a parameter for evaluation, as shown in Figure 2. The force at 50% displacement obviously increased with increasing PVP content, indicating an enhancement in the strength of the TS agglomerates. This result was in agreement with previous studies [5,28,29]. The PVP solution added could create a thin film (liquid bridge in the wet state) to induce an adhesion of starch granules, leading to the formation of capillary forces and strong solid bridges due to binder hardening after drying [21,30]. Thus, the addition of PVP, which acts as an agglomerating agent, could increase the particle strength of the TS agglomerates, which is dependent upon the concentration of PVP. In the case of the AMTS agglomerates, the 0.1 N AMTS agglomerates showed lower strength than the TS agglomerates. However, the particle strength of the AMTS agglomerates tended to increase with increasing HCl concentrations for starch modification. The 1.0 N AMTS agglomerates with 4% PVP presented the highest particle strength. These results could be explained by the characteristics of the AMTS after using different acid concentrations of modification. The higher the HCl concentrations were, the greater the percent relative crystallinity of the AMTS. This phenomenon resulted in an increase in the powder compressibility of the AMTS [19]. Thus, the increase in acid concentration also brought about a greater particle strength of the AMTS agglomerates.

### 3.2. Molecular Interaction and Thermal Behavior of the Agglomerates

**Figure 3:**
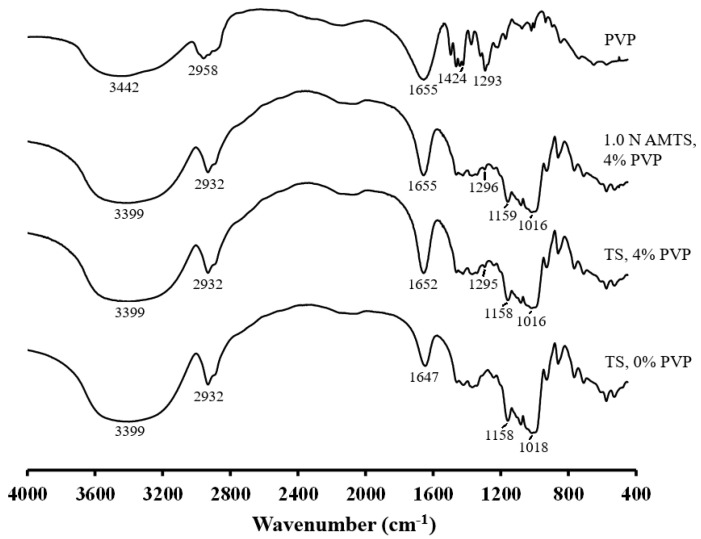
FTIR spectra of PVP, TS agglomerates, and AMTS agglomerates.

The FTIR spectrum of TS showed an O–H stretching peak at 3399 cm^−1^, C–H stretching peaks at 2932 cm^−1^, and O–H bending of water residues at 1647 cm^−1^, as presented in Figure 3. Moreover, the C–O stretching, C–C bending and O–H asymmetric stretching of the C–O–C glycosidic bond appeared at approximately 1018–1158 cm^−1^. The AMTS also presented the same FTIR spectrum as TS, which was reported previously [19]. The FTIR spectrum of PVP could be identified as follows: the O–H stretching at 3442 cm^−1^, the C–H stretching of the CH_2_ and ternary CH groups at approximately 2958 cm^−1^, the C=O stretching in the pyrrolidone group at 1655 cm^−1^, the CH deformation modes from the CH_2_ groups at approximately 1424 cm^−1^, and the C–N bending of the pyrrolidone structure at 1293 cm^−1^, which were similar to previous reports [31,32]. The TS and 1.0 AMTS agglomerates with 4% PVP gave a similar FTIR pattern. Two changes could be found when compared with those without PVP. The first was the shift of the O–H bending peak of the TS and 1.0 N AMTS agglomerates with 4% PVP to a higher wavenumber, suggesting that the water residues of the starch interacted with the carbonyl groups of PVP. This result confirmed hydrogen bond formation. Second, the small peak of C–N bending of PVP appeared at 1295–1296 cm^−1^ in the spectra of TS and 1.0 N AMTS agglomerates, although PVP was incorporated in a very small amount.

**Figure 4:**
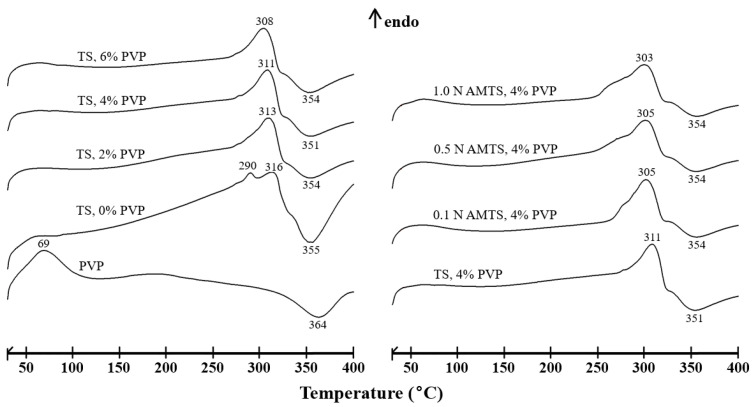
DSC thermograms of PVP, TS agglomerates, and AMTS agglomerates.

The DSC thermograms of the TS and AMTS agglomerates are shown in Figure 4. The TS agglomerates without PVP presented two endothermic peaks at 290 and 316 °C, indicative of the phase transition or melting of the amorphous region in the starch lamellae structure [19]. The exothermic degradation peak was also found at 355 °C. PVP presented a melting peak at 69 °C, followed by a degradation peak at 364 °C. The addition of PVP caused a single endothermic peak of the TS agglomerates, which moved to a lower temperature with increasing PVP content. The degradation temperature of the TS agglomerates with PVP was over the range of 351–354 °C. Moreover, no PVP melting peak appeared. This may be due to the small amount of PVP added. However, the thin film of PVP on the surface of the starch granules could possibly melt, inducing the phase transition of the starch lamellae structure. In addition, PVP molecules interacted with water residues in the starch granules, which was revealed by FTIR study and influenced this transition at higher temperatures. Apart from the TS agglomerates, the DSC thermograms of the AMTS presented two endothermic peaks at approximately 255–309 °C and the degradation peak at 354–357 °C that was reported previously [19], similar to that of the TS agglomerates without PVP. The phase transition temperature of the AMTS agglomerates was lower than that of the TS agglomerates. The change in the internal structure of the starch granules after acid hydrolysis may bring about the phase transition at lower temperatures induced by the same amount of PVP. Therefore, these findings indicated that the FTIR pattern and thermal behavior of the TS and AMTS agglomerates could be changed when PVP was added.

### 3.3. Flowability and Compressibility of the Agglomerates

Carr’s index and repose angle were used to evaluate the particle flowability of the agglomerates in this study, which are listed in Table 1. The bulk and tapped densities of the agglomerates, as shown in Table 1, were used to calculate the percent Carr’s index. All of the agglomerates had bulk density values in the range of 0.45–0.49 g cm^−^^1^, and the highest tapped density was found in the TS agglomerates without PVP, resulting in the highest Carr’s index of 34.41%. This value presented a flowability at a very poor level [33]. An increase in percent PVP incorporation led to a decrease in Carr’s index and the flowability, which was met to a good level when using PVP up to 4–6%. The Carr’s index values of the AMTS agglomerates using 4% PVP were over the range of 18.8–19.9%, which falls into a fair level of flowability. For the angle of repose, the TS agglomerates without PVP showed the highest value of 42.82°, which reflected passable flowability [34]. In contrast, the angle of repose values of all agglomerates with PVP were approximately 35°, suggesting good flowability.

Carr’s index value reflects the cohesiveness and the friction of powders under static conditions [35], whereas a dynamic friction property of powders can be determined using the angle of repose [34]. From these results, agglomeration of the TS using PVP could reduce the static and dynamic friction between the particles, leading to enhancement of particle flowability. In the case of AMTS, the AMTS in the particle size range of 75–150 µm using various HCl concentrations showed poor flowability, which was reported previously [19], because the irregular shape and surface roughness of the particles provided high interparticulate friction forces. The agglomeration using PVP in this study markedly decreased the Carr’s index and repose angle, suggesting that the thin film of PVP coated on the starch granule surface may reduce the interparticle friction force of the particles. Furthermore, the size enlargement and strong particle strength of the AMTS agglomerates improved the particle flowability.

**Figure 5:**
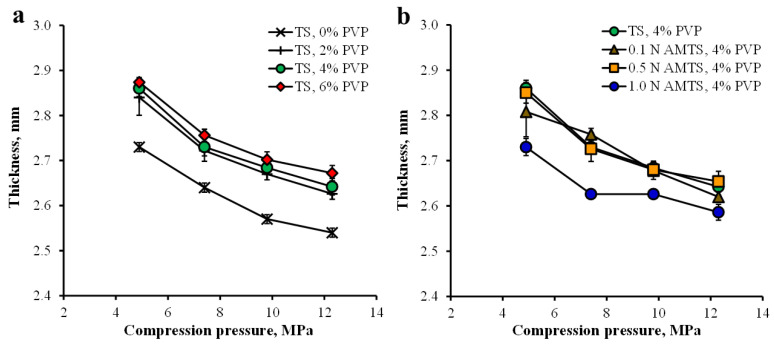
Thickness-compression pressure profiles of TS agglomerates using various concentrations of PVP (**a**), and AMTS agglomerates using 4% PVP (**b**). Each point is the mean ± S.D., *n* = 5.

**Figure 6:**
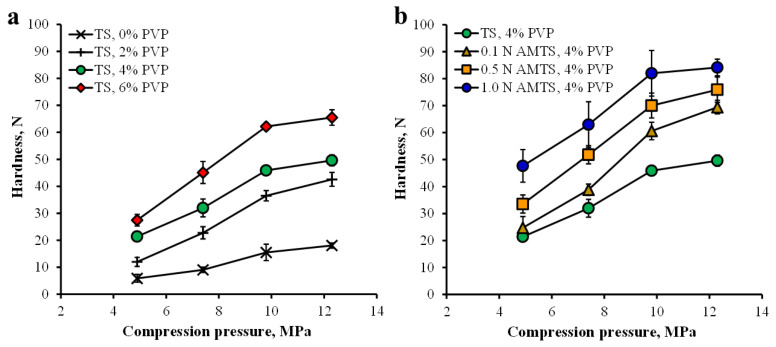
Hardness-compression pressure profiles of TS agglomerates using various concentrations of PVP (**a**), and AMTS agglomerates using 4% PVP (**b**). Each point is the mean ± S.D., *n* = 5.

The TS and AMTS agglomerates without drug were compressed at various pressures, and the thickness and hardness of the tablets were investigated. The TS agglomerates without PVP presented the lowest thickness tablets (2.73 ± 0.01 mm, *n* = 5), whereas the thicknesses of the tablets prepared from the TS agglomerates with PVP were in the range of 2.84–2.87 mm at a pressure of 4.9 MPa (Figure 5a). Moreover, the 1.0 N AMTS agglomerates displayed the lowest thickness tablets, which were found to be 2.73 ± 0.02 mm, *n* = 5, when compared with the other AMST agglomerate tablets that provided thicknesses of 2.80–2.85 mm (Figure 5b). An increase in pressure caused a decrease in the thicknesses of the tablets prepared from the TS and AMTS agglomerates (Figure 5). The hardness-compression profiles of the TS and AMTS agglomerate tablets are shown in Figure 6. The TS agglomerates without PVP gave the lowest tablet hardness, and the tablet hardness increased with increasing PVP content used for agglomeration (Figure 6a). Moreover, the tablet hardness of the TS agglomerates continuously increased as the compression increased. For the AMTS agglomerates, the AMTS agglomerates gave obviously higher tablet hardness than the TS agglomerates with the same PVP content (Figure 6b). Furthermore, the HCl concentration used directly influenced the tablet hardness, and the hardness of the tablets was also dependent upon the compression.

**Figure 7:**
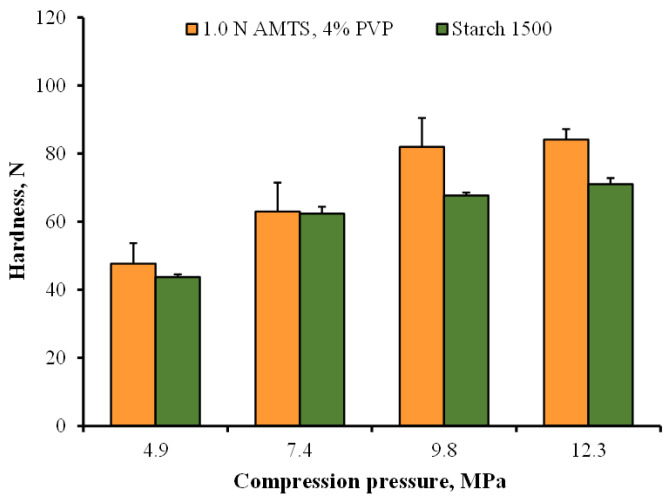
Tablet hardness of Starch 1500^®^ and 1.0 N AMTS agglomerates compressed at different pressures. Each value is the mean ± S.D., *n* = 5.

The flowability and compressibility of the 1.0 N AMTS agglomerates with 4% PVP were compared with Starch 1500^®^, which is a partially pregelatinized maize starch and has been used as a direct compression diluent for tablets [36]. The Carr’s index and angle of repose of Starch 1500^®^ were found to be 16.79 ± 0.28% and 37.62 ± 0.62° (*n* = 3), respectively. The results suggested fair-good flowability of Starch 1500^®^, which was similar to that of the 1.0 N AMTS agglomerates (Table 1). The characteristics of Starch 1500^®^ tablets without drug were also investigated. The thicknesses of Starch 1500^®^ tablets compressed using various pressures were in the range of 2.45–2.76 mm, which were not different from those of the 1.0 AMTS agglomerate tablets. The hardness of the starch 1500^®^ and 1.0 AMTS agglomerate tablets compressed using different pressures is shown in Figure 7. The results presented that the tablets prepared using both fillers showed a comparable hardness at 4.9 and 7.4 MPa. However, the 1.0 AMTS agglomerates provided greater tablet hardness than Starch 1500^®^ when using 9.8 and 12.3 MPa. These results suggested that the 1.0 N AMTS agglomerates had comparable flowability and enhanced compressibility to Starch 1500^®^, especially when higher compression pressures were applied.

In general, the starches possessed plastic deformation under compression [37], and an increase in pressure brought about a larger contact area of cold welding between the starch granules, leading to higher tablet hardness. Incorporation of PVP could modify the particle surface by adsorption and could promote cold welding (chemical bonds or solid bridges formed on the solid surface of the particles [38]) and plastic deformation of the agglomerates under compression [5]. The AMTS presented greater tablet hardness than the native starch in a previous study [19] due to the higher percent relative crystallinity of the AMTS, which directly affected the hardness of the compressed tablets prepared using starches modified by acid hydrolysis [14,19]. The higher acid concentrations used for TS modification caused a greater percent relative crystallinity of the AMTS, and a higher tablet hardness was also found [19]. The results in this study showed that the AMTS agglomerates with 4% PVP provided remarkably greater tablet hardness than the AMTS without PVP at the same HCl concentration used that was reported previously [19]. These findings indicated that the size enlargement of TS and AMTS by agglomeration using PVP could enhance particle flowability and promote the cold welding mechanism under compression, leading to better tablet characteristics, especially tablet hardness.

### 3.4. Characteristics of Drug-Loaded Tablets

The 40 mg PPN-loaded tablets compressed at 12.3 MPa were prepared, and the characteristics of the tablets are listed in Table 2. The thicknesses of all tablets were approximately 2.6 mm. The TS agglomerates without PVP gave the lowest hardness of tablets, whereas the TS agglomerate tablets presented greater hardness with increasing PVP contents. The tablets prepared using the 1.0 N AMTS agglomerates showed higher hardness than those using 0.1 N and 0.5 N AMTS agglomerates. Moreover, it could be observed that loading 40 mg PPN resulted in lower tablet hardness than the tablets without drug because drug particles could obstruct the cold welding of the TS and AMTS agglomerates, leading to a reduction in tablet hardness, which was similar to previous reports [39]. The TS agglomerate tablets without PVP showed the shortest disintegration time in both 0.1 N HCl and pH 6.8 phosphate buffer (Table 2). The incorporated PVP resulted in a longer disintegration time that was dependent upon the PVP content. These results were because of the higher tablet hardness when increasing PVP contents. The AMTS agglomerate tablets presented longer disintegration times than the TS agglomerate tablets with PVP in both media, but the disintegration time of the AMTS agglomerate tablets was not related to the HCl concentration used for modification.

**Figure 8:**
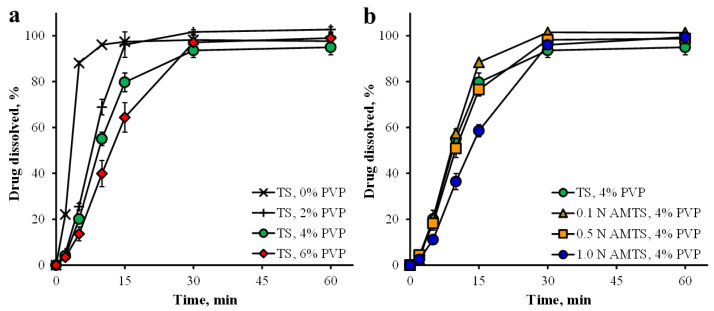
PPN dissolution profiles of tablets prepared by TS agglomerates (**a**), and AMTS agglomerates (**b**) in 0.1 N HCl. Each point is the mean ± S.D., *n* = 3.

**Figure 9:**
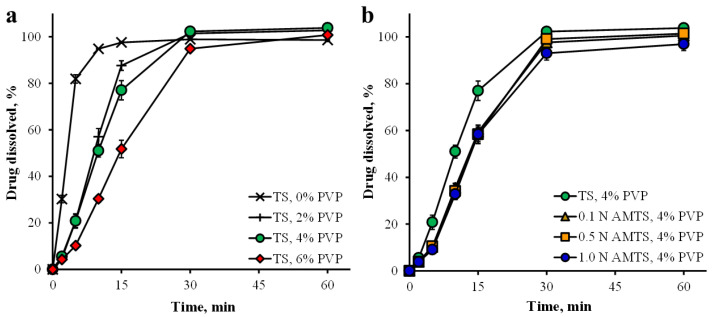
PPN dissolution profiles of tablets prepared by TS agglomerates (**a**), and AMTS agglomerates (**b**) in pH 6.8 phosphate buffer. Each point is the mean ± S.D., *n* = 3.

For the PPN dissolution test, the drug dissolution profiles in 0.1 N HCl are presented in Figure 8. Complete drug dissolution was found within 30 min of the test in acidic medium, and a similar result was also obtained in neutral medium (Figure 9). The slowest drug dissolution of the TS agglomerate tablets with 6% PVP could be observed in Figure 8a. Additionally, a longer T_50__%_ of the TS agglomerate tablets was found when increasing PVP contents (Table 2), suggesting that higher tablet hardness affected the drug dissolution of these tablets. The 1.0 N AMTS agglomerate tablets provided the slowest drug dissolution and the longest T_50__%_, as shown in Figure 8b and Table 2, respectively. This result was influenced by the highest tablet hardness in the AMTS agglomerates used. In addition, the dissolution medium used in this study affected the disintegration time and drug dissolution rate of the agglomerate tablets (Figure 9a,b). The slightly longer disintegration time and the slightly slower drug dissolution rate of the tablets in pH 6.8 phosphate buffer were obtained when compared with using 0.1 N HCl, which was similar to a previous report [19].

### 3.5. Use of the AMTS Agglomerates as Direct Compression Fillers

**Figure 10:**
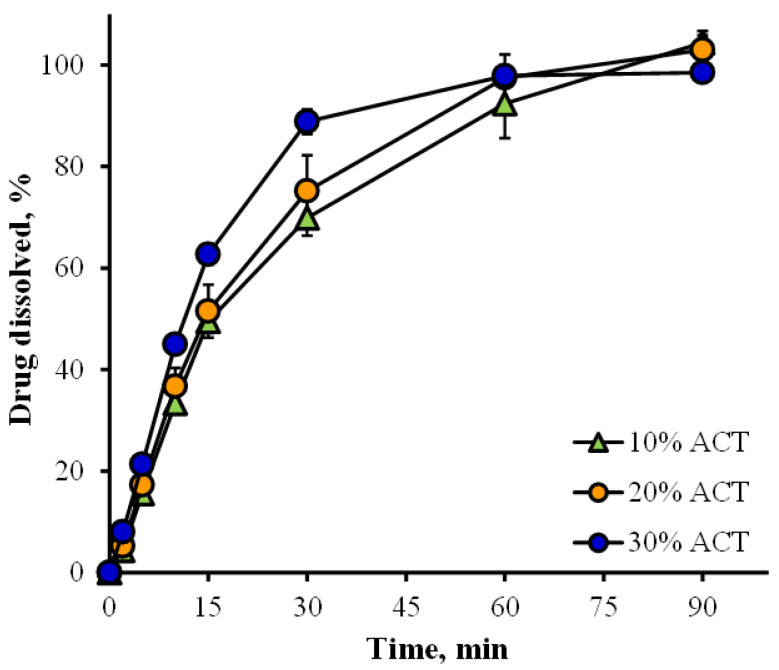
ACT dissolution profiles of the 1.0 N AMTS agglomerate tablets containing various contents of ACT. Each point is the mean ± S.D., *n* = 3.

The 1.0 N AMTS agglomerates were selected and employed as a direct compression filler because of their good flow capacity and compressibility, and the dilution potential or carrying capacity of these agglomerates was investigated in this study. Dilution potential or carrying capacity was demonstrated as the highest percentage of noncompressible drug in the acceptable tablets [4]. ACT was the model drug in this study because it had a high capping tendency [40], and the compressed tablets of pure ACT also had very low hardness [41,42]. Additionally, the Carr’s index and repose angle of acetaminophen powder were found to be 45.15 ± 1.65% and 60.19 ± 0.75° (*n* = 3), respectively, indicative of very poor flowability with high cohesiveness. The results showed that no capping of the tablets occurred, and the mean thickness of all tablets was in the range of 3.42–3.47 mm (Table 3). The weights of the tablets with 0–30% *w*/*w* ACT showed a low variation, with RSD less than 2%, whereas the tablets with 40% ACT had the highest RSD, which was found to be 4.73%. However, the range of ACT content in the tablets was 98.11–101.45%. The hardness of the tablets decreased with increasing percent ACT added, leading to a higher percent friability and shorter disintegration time in 0.1 N HCl. These results suggested that an ACT content greater than 30% added to the 1.0 N AMTS agglomerates resulted in an increase in the cohesiveness of the mixtures, leading to a reduction in powder flowability, and a higher tablet weight variation was then obtained. Furthermore, the increase in ACT powder content could obstruct cold welding of the AMTS agglomerates, resulting in a decrease in tablet hardness, an increase in percent friability and a shortening of disintegration time. The ACT dissolution profiles of the tablets containing 10 to 30% ACT in 0.1 N HCl are displayed in Figure 10. The drug dissolution of the tablets was completed within 90 min. The T_50__%_ values of the tablets loaded with 10, 20, and 30% ACT were 16.00 ± 1.59, 15.53 ± 2.23, and 11.43 ± 0.43 min (*n* = 3), respectively. The faster dissolution of the tablets with 30% ACT was obtained due to shorter disintegration time and a higher ratio of drug when compared with the agglomerates used. For the dilution potential study, it was suggested that the 1.0 N AMTS agglomerates had good carrying capacity such that a drug with poor flowability and compressibility could be incorporated at less than 30% to achieve acceptable tablets.

### 3.6. Stability Study of the Tablets

The tablets prepared using 20% ACT in 1.0 N AMTS agglomerates (70 mg ACT in 350 mg tablets) were used in this study. The purpose of this study was to investigate the change in tablets using 1.0 N AMTS agglomerates as the main diluent when exposed to high temperature and humidity. ACT was suitable for this study because it is stable at 45 °C in the form of pure powder [43] and showed good physical and chemical stability in the form of tablets when stored at 40 °C/75% RH [44]. The characteristics of the tablets stored under different conditions for 3 months compared with the tablets at the initial stage are listed in Table 4. The appearance of the tablets showed a white color, and no change in color was observed when kept under different conditions for 3 months. However, the tablets kept under the accelerated condition showed slightly greater thickness and obviously higher hardness than those at the initial stage and other storage conditions. Veronica et al. [45] reported that storage of TS under conditions of increasing RH resulted in a gradual increase in granule size due to the moisture absorption capacity of starches. This result indicated that the AMTS used as a filler could absorb moisture under high RH conditions, leading to an increase in the tablet thickness. The hardness of tablets using PVP as a binder increased after storage under 40 °C/52% RH, which was reported by Sarisuta and Parrott [46]. This phenomenon could be explained by the fact that the water molecules may enhance the interparticle bonding of the AMTS agglomerated using PVP in the tablets, which may increase the tablet hardness. The ACT contents in the tablets stored under different conditions for 3 months were in the range of 98.33–99.35%, which was not different from that of the tablets in their initial stage. The disintegration time and drug dissolution were tested in 0.1 N HCl as a medium. The disintegration time of the tablets stored under different conditions was slightly longer than that at the initial stage. Accelerated conditions resulted in the longest disintegration time, which was due to an obvious increase in tablet hardness. The disintegration time results were not correlated with the dissolution rate of ACT. T_50__%_ of the tablets presented faster drug dissolution when stored under accelerated conditions, and the tablets stored both under ambient conditions and in refrigerators presented slower dissolution of ACT than the initial stage. However, the tablets kept under the three conditions showed similarity factor (f_2_) values between 50 and 100, suggesting the similarity of ACT dissolution profiles in acidic medium. Thus, it is important to control the temperature and humidity of the storage environments to maintain the long-term stability of the drug-loaded AMTS agglomerate tablets.

## 4. Conclusions

Particle size enlargement of TS and AMTS can be successfully performed by agglomeration using PVP. The thermal behavior of the TS and AMTS agglomerates changes at high temperature due to the induction of melted PVP, and PVP molecules can interact with the water residues of the starch via hydrogen bonding. The higher PVP concentrations used provide greater particle strength of the TS agglomerates, and increasing the acid concentration for starch modification enhances the strength of the AMTS agglomerates. The TS and AMTS agglomerates present good particle flowability, whereas the AMTS agglomerates display higher tablet hardness than the TS agglomerates. The addition of PVP can extend the disintegration time and slow drug dissolution from the TS agglomerates. In addition, the 1.0 N AMTS agglomerate tablets had the longest disintegration time and the slowest drug dissolution when using 4% PVP, resulting in the highest tablet hardness. For stability of the AMTS tablets, the accelerated condition causes greater thickness and hardness of the AMTS tablets after storage for 3 months, but slightly affects the disintegration time and drug dissolution when compared with the tablets stored under ambient conditions and in the refrigerator. The AMTS agglomerates display good dilution potential or carrying capacity and provide desirable characteristics of tablets prepared by the direct compression method. However, the potential impact of the botanical resources of TS on the mechanical properties of the agglomerates should be concerned.

## Figures and Tables

**Table 1:** Characteristics and particle flowability of TS and AMTS agglomerates.

Starches	PVP (% *w*/*w*)	Yield (% *w*/*w*)	Moisture Content ^a^ (% *w*/*w*)	Bulk Density ^a^ (g cm^−^^3^)	Tapped Density ^a^ (g cm^−^^3^)	Carr’s Index ^a^ (%)	Angle of Repose ^a^ (°)
TS	0	34.88	12.06 ± 0.16	0.46 ± 0.01	0.70 ± 0.01	34.41 ± 0.22	42.82 ± 0.21
	2	46.21	9.92 ± 0.25	0.48 ± 0.01	0.58 ± 0.01	17.71 ± 0.17	35.65 ± 0.73
	4	56.86	9.43 ± 0.37	0.45 ± 0.01	0.52 ± 0.01	14.08 ± 0.14	35.86 ± 0.36
	6	55.37	9.42 ± 0.14	0.46 ± 0.01	0.54 ± 0.01	13.91 ± 0.06	35.33 ± 0.36
0.1 N AMTS	4	55.37	9.08 ± 0.23	0.49 ± 0.01	0.60 ± 0.01	18.80 ± 0.12	35.17 ± 0.23
0.5 N AMTS	4	63.66	9.34 ± 0.48	0.47 ± 0.01	0.59 ± 0.01	19.97 ± 0.58	35.27 ± 0.28
1.0 N AMTS	4	72.63	9.51 ± 0.27	0.45 ± 0.02	0.56 ± 0.03	19.88 ± 0.42	35.42 ± 0.84

^a^ Data are mean ± S.D., *n =* 3.

**Table 2:** Characteristics of PPN tablets prepared using TS and AMTS agglomerates.

Starches	Thickness ^a^ (mm)	Hardness ^a^ (N)	0.1 N HCl ^b^		pH 6.8 Phosphate Buffer ^b^	
			DT (min)	T_50__%_ (min)	DT (min)	T_50__%_ (min)
TS, 0% PVP	2.62 ± 0.01	5.69 ± 0.96	0.47 ± 0.01	3.27 ± 0.02	0.50 ± 0.01	3.15 ± 0.03
TS, 2% PVP	2.60 ± 0.01	37.85 ± 7.51	2.17 ± 0.01	7.84 ± 0.27	2.68 ± 0.07	9.04 ± 0.48
TS, 4% PVP	2.65 ± 0.02	44.72 ± 8.45	2.47 ± 0.01	9.31 ± 0.36	2.98 ± 0.03	9.86 ± 0.51
TS, 6% PVP	2.63 ± 0.02	52.96 ± 1.64	4.04 ± 0.01	12.10 ± 1.21	4.61 ± 0.18	14.73 ± 0.83
0.1 N AMTS, 4% PVP	2.68 ± 0.02	43.35 ± 3.48	3.28 ± 0.11	8.97 ± 0.29	3.77 ± 0.01	13.02 ± 0.32
0.5 N AMTS, 4% PVP	2.66 ± 0.01	42.17 ± 2.97	4.22 ± 0.03	9.93 ± 0.62	4.75 ± 0.16	13.37 ± 0.55
1.0 N AMTS, 4% PVP	2.66 ± 0.01	54.59 ± 9.45	4.19 ± 0.04	13.04 ± 0.68	4.68 ± 0.07	14.73 ± 0.39

^a^ Data are mean ± S.D., *n* = 5; ^b^ Data are mean ± S.D., *n* = 3; DT = disintegration time.

**Table 3:** Physical properties of tablets prepared using 1.0 AMTS agglomerates as direct compression fillers.

ACT Added in Mixture (% *w*/*w*)	Thickness ^a^ (mm)	Tablet Weight ^b^ (mg)	Hardness ^a^ (N)	Friability (%)	ACT Content in Tablets ^c^ (%)	DT ^c^ (min)
0	3.44 ± 0.01	353.26 ± 1.34 (%RSD = 0.38)	69.14 ± 2.76	0.48	-	5.86 ± 0.01
10	3.45 ± 0.01	349.59 ± 2.29 (%RSD = 0.66)	69.46 ± 2.22	0.77	100.78 ± 0.38	7.34 ± 0.12
20	3.46 ± 0.02	351.42 ± 3.44 (%RSD = 0.98)	71.20 ± 2.02	0.83	98.11 ± 0.42	6.49 ± 0.12
30	3.47 ± 0.03	355.64 ± 5.75 (%RSD = 1.62)	46.58 ± 3.61	1.19	100.83 ± 0.79	4.76 ± 0.23
40	3.42 ± 0.06	349.59 ± 16.53 (%RSD = 4.73)	41.51 ± 7.33	1.92	101.45 ± 1.30	3.63 ± 0.52

^a^ Data are mean ± S.D., *n* = 5; ^b^ Data are mean ± S.D., *n* = 20; ^c^ Data are mean ± S.D., *n* = 3; RSD = relative standard deviation.

**Table 4:** Stability study of ACT tablets prepared using 1.0 AMTS agglomerates as direct compression fillers.

Condition	Thickness ^a^ (mm)	Hardness ^a^ (N)	ACT Content ^b^ (%)	DT ^b^ (min)	T_50__%_^b^ (min)	Similarity Factor (f_2_)
Initial	3.46 ± 0.02	71.20 ± 2.02	98.11 ± 0.42	6.49 ± 0.12	15.55 ± 2.23	-
3 months storage						
Ambient condition 26 ± 2 °C, 52 ± 3% RH	3.55 ± 0.03	80.02 ± 2.31	98.33 ± 3.99	6.62 ± 0.13	20.93 ± 3.00	56.60
Accelerated condition 45 ± 1 °C, 75% RH	3.60 ± 0.05	136.15 ± 9.44	99.35 ± 4.08	7.65 ± 0.20	12.95 ± 1.37	68.75
Refrigerator 2–8 °C, 20 ± 2% RH	3.52 ± 0.03	79.43 ± 2.06	99.23 ± 3.29	7.19 ± 0.40	17.15 ± 2.45	70.43

^a^ Data are mean ± S.D., *n* = 5; ^b^ Data are mean ± S.D., *n* = 3; DT = disintegration time.

## Data Availability

Data are contained within the article.
